# Shape unrestricted topological corner state based on Kekulé modulation and enhanced nonlinear harmonic generation

**DOI:** 10.1515/nanoph-2024-0116

**Published:** 2024-06-03

**Authors:** Kai Guo, Huiyuan Wang, Jiawei Xiong, Jintao Wu, Bingyi Liu, Zhongyi Guo

**Affiliations:** School of Computer and Information, 12513 Hefei University of Technology, Hefei 230009, China; Key Lab of Modern Optical Technologies of Education Ministry of China, Soochow University, Suzhou 215006, China

**Keywords:** topological photonics, nonlinear optics, topological corner states

## Abstract

Topological corner states have been extensively utilized as a nanocavity to increase nonlinear harmonic generation due to their high *Q*-factor and robustness. However, the previous topological corner states based nanocavities and nonlinear harmonic generation have to comply with particular spatial symmetries of underlying lattices, hindering their practical application. In this work, we design a photonic nanocavity based on shape unrestricted topological corner state by applying Kekulé modulation to a honeycomb photonic crystal. The boundaries of such shape unrestricted topological corner state are liberated from running along specific lattice directions, thus topological corner states with arbitrary shapes and high *Q*-factor are excited. We demonstrate enhancement of second (SHG) and third harmonic generation (THG) from the topological corner states, which are also not influenced by the geometry shape of corner. The liberation from the shape restriction of corner state and nonlinear harmonic generation are robust to lattice defects. We believe that the shape unrestricted topological corner state may also find a way to improve other nonlinear optical progress, providing great flexibility for the development of photonic integrated devices.

## 1 Introduction

Topological states have been extensively investigated in not only condensed matter physics [1], [2], [3] but also in classical physics [4], [5], [6], such as acoustics [7], [8], [9], [10], [11], [12], [13], [14] and optics [15], [16], [17], [18], [19], [20], [21], [22], [23], because of their excellent topological protection characteristics. Very recently, higher-order topological insulators (HOTIs) have attracted a huge amount of research attentions since they break the conventional bulk-edge correspondence and support topological states of two or more dimensions lower than them [23], [24], [25], [26], [27]. Among them, two-dimensional photonic HOTI have been deeply studied [23], [24], [28], in which the zero-dimensional corner state could exist with several excellent properties such as topological robustness and high quality factor, indicating its promise as an excellent nanocavity [29], [30], [31], [32], [33]. It is these properties, as well as their subwavelength structural units, that make topological states useful in many fields, such as lasers [30], near-field imaging [34], [35], [36] and on-chip optical information processing [37].

As the two general issues in optics, topology and nonlinearity are combined in photonics, becoming a hot topic [34], [36], [[38], [39], [40], [41], [42], [43]]. It has been demonstrated that the efficiencies of the optical nonlinearity could be greatly enhanced with the help of the edge and corner states in topological photonic structures in comparison to the bulk state and the dielectric slab without any photonic structures [41], [42], [44], [45], [46], [47]. Besides, the edge and corner states provide us an opportunity to artificially engineer the frequency conversion process with topological robustness [41], [44], [45]. These results may bridge nonlinear optics with topological physics for the integrated nanophotonic circuit with excellent robustness. Specifically, the corner states contributing enhancement of optical nonlinearity is crucial for on-chip optical information technology which requires highly efficient photon–photon interaction. Among them, second (SHG) [42], [44], [45] and third harmonic generation (THG) [36], [43] enhancement from corner states have been extensively investigated since their high efficiencies are relatively high in comparison to high orders and the phase-matching requirements are easily satisfied.

However, in most of the reported photonic HOTIs, which are characterized with either quantized quadrupole momentum [48] or quantized dipole momentum [23], the two edges of a topological corner state should walk along fixed lattice directions due to the specific spatial symmetries of the underlying unit-cells [49]. For example, there are only a limited choices for angles of corners, such as 90° and 60° for square [24], [29], [32] and Kagome [26] lattices, respectively. From the perspectives of both fundamental physics and practical applications, it greatly hinders the development of HOTIs. In fact, the previous scheme usually relies on two discrete phase values (0 and *π*) of the Dirac gap (mass terms of the Hamiltonian) [19]. Recently, a topological cavity utilizes a complete 2*π* vortex phase of Dirac-mass to confine photons in-plane, i.e. a topological zero modes induced by Dirac vortices [50], [51]. It has been proposed that by introducing phase vortex at the corners of an acoustic HOTI through Kekulé modulation can help corner states break away from the limitation of lattice shape, i.e. topological corner states with arbitrary shapes can be excited theoretically [52], [53], [54]. This reduction of restrictive condition of the corner shape may promote the development of optical nonlinearity in topological photonics to a higher level.

In this work, we use Kekulé modulation to obtain the photonic HOTIs and demonstrate the enhancement of nonlinear harmonic generation from the Dirac-vortices based corner states with arbitrary shapes. The Kekulé modulation introduces a complete 2*π* vortex phase to various shaped corners, ensuring the definitely existence of topological corner states which no longer comply with specific spatial symmetries of underlying lattices. Numerical simulations demonstrated that the corner states based on Kekulé modulation exhibit high-density local field with high quality factor. To show the advantage of the designed photonic HOTI, we utilize these arbitrary shaped corner states to enhance SHG and THG. Finally, the robustness of topological corner states and the stability of the enhanced nonlinear harmonic generations are validated by introducing several defects. Such nonlinear harmonic generations from shape unrestricted corner states may greatly improve the flexibility of topology devices.

## 2 Results and discussion

### 2.1 Design of the system

**Figure 1:**
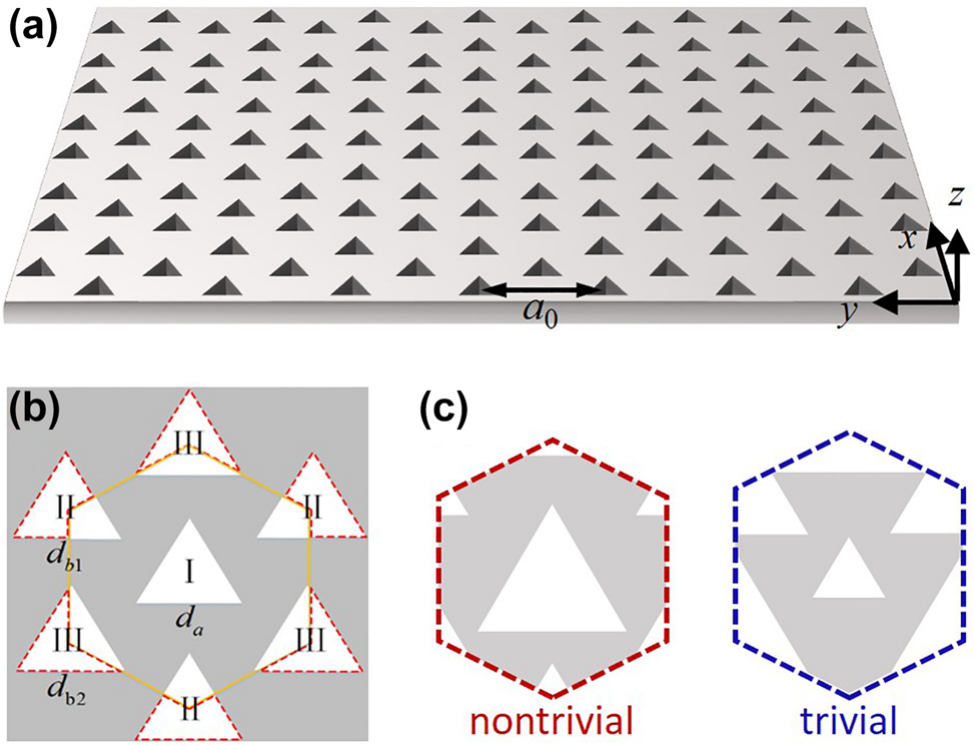
Schematic of initial photonic crystal. (a) A dielectric slab with equilateral-triangular air-holes arranged in a triangular lattice. We assume the slab is infinite in *z*-direction. Lattice constant: *a*_0_ = 1 μm. (b) A single lattice structure. *d*_*a*_, *d*_*b*1_ and *d*_*b*2_ denotes side length of the triangular-holes I, II and III, respectively. (c) Nontrivial and trivial patterns of a supercell.

Figure 1(a) schematically illustrates the overall diagram of the designed two-dimensional photonic crystal, which is made of dielectric slab with equilateral-triangular air-holes arranged in a triangular lattice. The gray and white regions indicate the dielectric slab and air-holes, respectively. The slab is assumed to be infinite in *z*-direction. Initially, all triangular air-hole structures are same. When we calculate the bands of the primitive unit cell, the *C*_3*v*_ symmetry guarantees Dirac cones emerging at corners *K*_±_ of the first Brillouin zone (FBZ) [55], [56]. When we consider an enlarged unit cell (marked by the orange line and shown in the zoom-in inset), the inequivalent Dirac cones at valleys *K*_±_ will be folded back and a double Dirac cone emerges at Γ point [49], [57]. As shown in Figure 1(b), the parts inside the red dotted line belong to the adjacent cells. The side lengths of the triangular-holes I, II and III are *d*_*a*_, *d*_*b*1_ and *d*_*b*2_, respectively. The lattice constant is *a*_0_ = 1 μm.

Herein, a Kekulé texture is imposed on the designed photonic crystal to alternate the coupling strength between adjacent sublattice and implement intervalley coupling, which is analogy to the alternating arrangement of single and double bonds in the benzene structural formula [58]. We set the side length of the triangle centered at **r** = (*x*, *y*) as

(1) $$d\left(r\right)=d_{0}+\delta d\cos\left(K\cdot r+\varphi \right)$$

*d*_0_ is the initial side length, **K** = **K**_+_ − **K**_−_ is the Kekulé vector, *δd* and *φ* represents the strength and phase of the modulation. After imposing the Kekulé modulation, the side lengths of the triangular-holes become *d*_*a*_ = *d*_0_ + *δd*cos(*φ*), *d*_*b*1_ = *d*_0_ + *δd*cos(*φ* + 2*π*/3) and *d*_*b*2_ = *d*_0_ + *δd*cos(*φ* + 4*π*/3). Regarding the frequency range of interest, *d*_0_ and *δd* as *a*_0_/1.16 and *a*_0_/1.9, respectively. The enlarged lattices corresponding to *φ* = 0 and *φ* = *π* could be classified as topological nontrivial and trivial cases, respectively, as schematically shown in Figure 1(c).

To study the optical responses, a two-dimensional HOTI made of AlGaAs slab is considered. The refractive index of AlGaAs and air is set as *n* = 3.0288 and *n* = 1, respectively. The second- and third-order nonlinear susceptibility tensors of AlGaAs contain non-zero components in the non-diagonal positions, i.e. the nonlinear responses are dependent on the crystalline structure of AlGaAs [59], [60], [61]. We focus on the transverse magnetic (TM) mode with out-of-plane electric field *E*_*z*_ and in-plane magnetic field *H*_*x*,*y*_. For simplicity, the AlGaAs is assumed with specific crystalline structures for SHG and THG. The second-order nonlinear frequency conversion is conducted in AlGaAs with [111] crystalline structure, which is described by $P_{z}^{\text{SHG}}=\varepsilon_{0}\chi^{\left(2\right)}E_{z}^{2}$ with *χ* ^(2)^ = 1.0 × 10^−10^ m/V [60], [62]. The third-order nonlinear frequency conversion is conducted in AlGaAs with [001] crystalline structure, which is described by $P_{z}^{\text{THG}}=\varepsilon_{0}\chi^{\left(3\right)}E_{z}^{3}$ with *χ* ^(3)^ = 2.6 × 10^−18^ m^2^/V^2^ (the typical value of *χ* ^(3)^ varies from 1.6 × 10^−17^ m^2^/V^2^ to 6.2 × 10^−19^ m^2^/V^2^) [61], [62]. *ɛ*_0_ is the permittivity of vacuum. All numerical full-wave simulations are performed in the COMSOL multiphysics software. It is worthy to note that the second- and third-order nonlinear processes are not calculated simultaneously.

### 2.2 Topological properties of the system

**Figure 2:**
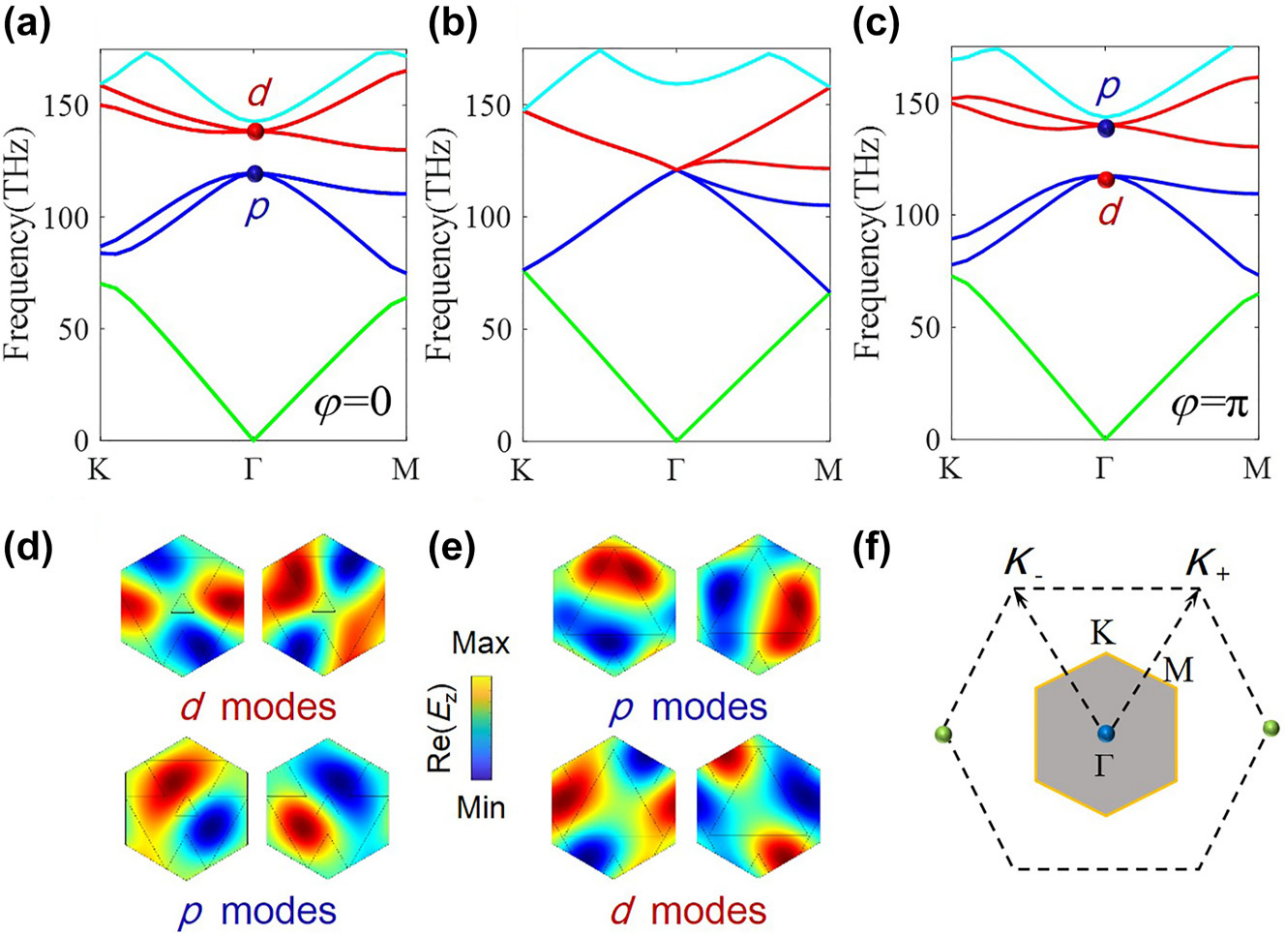
Band dispersion and mode patterns of the photonic crystal with homogeneous Kekulé modulations. (a) Band structure for *δd* = *a*_0_/1.9 and *φ* = 0, corresponding to trivial case. (b) Band structure for *δd* = 0, i.e. the initial case. (c) Band structure for *δd* = *a*_0_/1.9 and *φ* = *π*, corresponding to nontrivial case. In these three cases, *d*_0_ = *a*_0_/1.16. Eigenfield distributions of *E*_*z*_ at Γ points of both (d) trivial and (e) nontrivial cases. (f) The first irreducible Brillouin zones of the initial triangular lattice and the enlarged unit cell (gray hexagon). Two *K* valleys (at the green Dirac points) are folded onto Γ point, through coupling by the Kekulé vector **K** = **K**_+_ − **K**_−_.

Figure 2(a)–(c) plot the band structures of the photonic crystal with *δd* = *a*_0_/1.9, *φ* = 0 and *δd* = *a*_0_/1.9, *φ* = *π*, respectively, exhibiting the same dispersion feature. The band structures are numerically calculated by employing the eigenvalue module of the COMSOL multiphysics software. The blue and red lines represent double degeneracy bands. The eigenfields of *E*_*z*_ at Γ point (Figure 2(d) and (e)) show the band inversion between the dipole-like (*p*) and quadrupole-like (*d*) modes. The positions of *p* and *d* modes in the energy band judges that the modulation phase *φ* = 0 corresponds to trivial case, and *φ* = *π* makes the photonic crystal topological nontrivial, hosting pseudospin-Hall edge states. Figure 2(b) plots the band structure of the photonic crystal with *δd* = 0, and a four-fold degeneracy appears at the Γ point, which is guaranteed by the band-folding effect and *C*_6_ rotation symmetry. Figure 2(f) shows the first Brillouin zones of the initial and enlarged photonic crystals. The Kekulé lattice has a smaller Brillouin zone (gray hexagon) and rotated over *π*/6 with respect to the original Brillouin zone (dashed line).

A honeycomb-lattice photonic crystal with textured tight-binding hopping amplitude can be described by the Hamiltonian in the real space [50]

(2) $$H=\underset{r}{\sum }\overset{3}{\underset{l=1}{\sum }}\left(t+\delta t_{r,l}\right)a_{r}^{†}b_{r+s_{l}}+\text{H.c.}$$

where *δt*_**r**,*l*_ = 0 indicates the initial photonic crystal, and *a*_**r**_ and $b_{r+s_{l}}$ denote the annihilation operator on sublattice A of the unit cell at **r** and its neighboring sublattice B with a displacement **s**_*l*_, respectively. In the designed honeycomb-lattice, $s_{1}=\left(0,-\sqrt{3}a_{0}/3\right)$ , $s_{2}=\left(a_{0}/2,-\sqrt{3}a_{0}/6\right)$ , and $s_{3}=\left(-a_{0}/2,-\sqrt{3}a_{0}/6\right)$ . The homogeneous Kekulé modulation on the side length of the triangular-holes may introduce a bond texture in the tight-binding model, and the hopping amplitude can be approximately written as $t_{r,l}=t_{0}-2\delta t_{0}\cos\left[K\cdot \left(r+s_{l}\right)+\varphi \right]$ [52]. This hopping indicates intervalley couplings, i.e. the modes at **k** and **k** + **K** in the momentum space are coupled and folded onto the center of the superlattice Brillouin zone, as shown in Figure 2(f). After replacing the hopping in Eq. (2) with the modulated hopping, we adopt Fourier transform to the tight-binding Hamiltonian and obtain

(3) $$\begin{array}{c} H\left(k\right)=\varepsilon \left(k\right)a_{k}^{†}b_{k}+\frac{\delta t_{0}}{t_{0}}{\text{e}}^{\text{i}\varphi }\varepsilon \left(k+K\right)a_{k+K}^{†}b_{k}\\ +\frac{\delta t_{0}}{t_{0}}{\text{e}}^{-\text{i}\varphi }\varepsilon \left(k-K\right)a_{k-K}^{†}b_{k}+\text{H.c.} \end{array}$$

where $\varepsilon \left(k\right)=t_{0}\sum_{l=1}^{3}\exp\left(ik\cdot s_{l}\right)$ .

**Figure 3:**
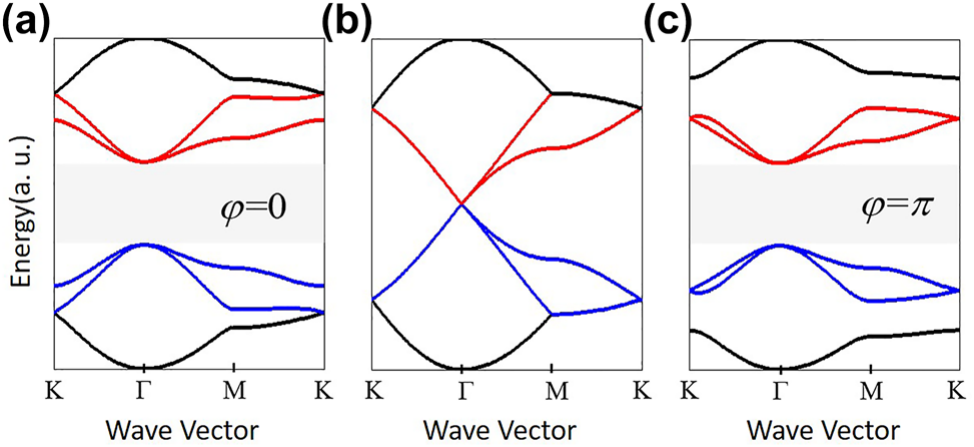
Energy dispersion calculated from the tight-binding Hamiltonian. (a) *δt*_0_ = 0.5 and *φ* = 0, (b) *δt*_0_ = 0, (c) *δt*_0_ = 0.5 and *φ* = *π*.

We calculate the energy dispersion of Eq. (3) for several typical values of *δt*_0_ and *φ*. When *δt*_0_ = 0, a four-fold degeneracy exists at the Γ point due to the band-folding effect, as shown in Figure 3(b). When *δt*_0_ ≠ 0, the band-folding effect is broken and the *C*_6_ symmetry will be reduced to *C*_3_ symmetry, resulting in a complete bandgap around Γ point. It is demonstrated by Figure 3(a)–(c) which plot the energy dispersion for the cases with *δt*_0_ = 0.5, *φ* = 0 and *δt*_0_ = 0.5, *φ* = *π*, respectively. The results in Figure 3 are in good agreements with those in Figure 2(a)–(c). The spin Chern number can be calculated as $C_{\text{s}}=\int_{\text{BZ}}\Omega \left(k\right)\text{d}S$ , where $\Omega \left(k\right)$ is the Berry curvature and BZ represents the whole Brillouin zone. For the case with *δt*_0_ = 0.5, *φ* = 0 and *δt*_0_ = 0.5, *φ* = *π*, the spin Chern number is $C_{\text{s}}^{\pm }=\pm 1$ and $C_{\text{s}}^{\pm }=\pm 0$ , respectively. ‘±’ represents the top and bottom edge of the bandgap around the Γ point. The values of the spin Chern number indicate their opposite topological phases, agreeing well with the results in Figure 2.

For the low energy spectrum around the Γ point, the six-band Hamiltonian can be reduced to a four-band effective Hamiltonian [50], [53],

(4) $$H\left(k\right)=\nu_{\text{D}}\tau_{0}⊗\left(k_{x}\sigma_{1}+k_{y}\sigma_{2}\right)+\left(m_{1}\tau_{1}-m_{2}\tau_{2}\right)⊗\sigma_{3}$$

where *k*_*x*_ and *k*_*y*_ are the momentums, *σ* and *τ* are both Pauli matrices. *m*_1_ and *m*_2_ are on behalf of intervalley couplings introduced by the Kekulé texture. The two mass terms form a complex Dirac mass *m* = *m*_1_ + *im*_2_, winding in plane as *m*(**r**) ∝ e^i*φ*(**r**)^ *δd*. Therefore, the Kekulé modulation opens a full band gap proportional to the value of *δd*, and it also brings in a phase vortex *φ* **r** for the Dirac mass, i.e. Dirac vortex, with negligible impact on the band gap size. It is worthy to note that this topological systems does not reply on two discrete phase values (0 and *π*) of the Dirac gap, where the topological corner states are characterized by either quantized quadrupole momentum [48] or quantized dipole momentum [23]. In contrast, the Kekulé modulation introduces complete 2*π* vortex phase through Dirac mass, and the winding number of the Dirac mass is the topological invariant of the vortex. This characteristic ensure emergence of topological corner state, which is independent on the geometry shapes.

### 2.3 Shape unrestricted corner states and nonlinear harmonic generation

**Figure 4:**
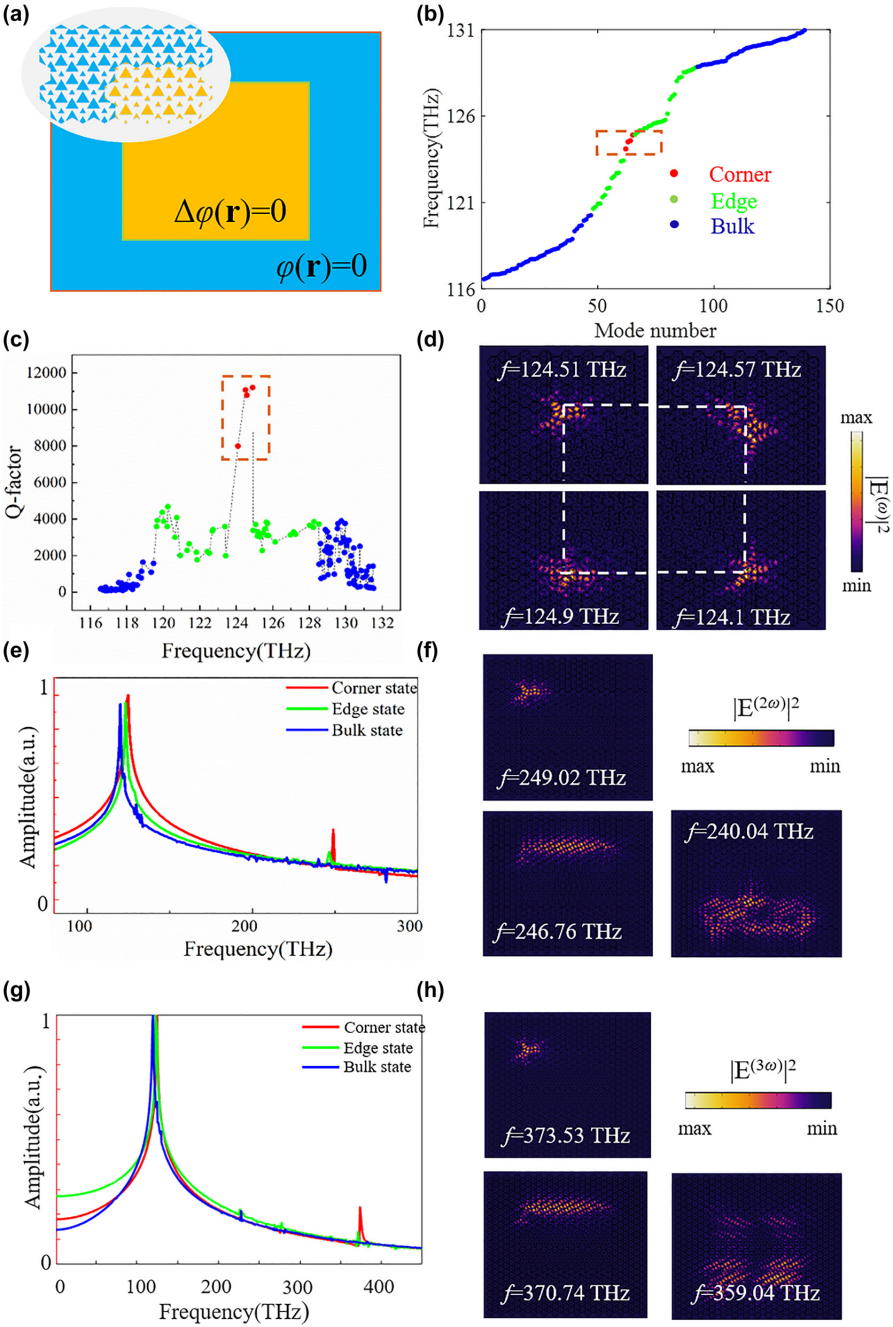
Corner states in a square HOTIs based on the Kekulé modulation and the enhanced nonlinear harmonic generatio. (a) Schematics of the constructed square HOTIs based on the Kekulé modulation. (b) Eigenmodes of the constructed HOTIs. (c) *Q* factor of the eigenmodes. (d) Electric field distributions of the topological corner states. (e) SHG frequency spectra obtained from the bulk, edge and corner state. (f) Electric field distributions of SHG corresponding to bulk, edge, and corner state. (g) THG frequency spectra obtained from the bulk, edge and corner state. (h) Electric field distributions of THG corresponding to bulk, edge, and corner state. The corner state in (g)–(h) indicates the upper-left corner.

To investigate, it is started by constructing a square domain of the above Kekulé modulated triangular lattice without regarding to its spatial symmetry, which is essential for many previous topological corner state in HOTIs [24], [26], [29], [32], [49]. As shown in Figure 4(a), the constructed HOTI could be divided into two different regions according to their modulation phases. The yellow square domain is fulfilled with Kekulé lattice, whose modulation phase $\varphi \left(r\right)$ is smoothly aperiodic and satisfies two-dimensional Laplace’s equation $\Delta \varphi \left(r\right)=0$ with the boundary condition of $\varphi \left(r\right)=0$ or $\varphi \left(r\right)=2\pi$ at the edges of the square domain. The surrounding blue lattice is with modulation phase of $\varphi \left(r\right)=0$ , serving as cladding. The inset schematically shows the constructed structure at the upper-left corner. Due to the continuity on the boundaries of the square domain, $\Delta \varphi \left(r\right)$ is uniquely determined by Eq. (1) when the structure is constructed. Around the four corners of the inner domain, the smooth profile introduce phase vortices, thereby guarantee existence of topological corner state.

The eigenmode simulation of the constructed square HOTI is studied and the results are plotted in Figure 4(b). The red, green and blue circles represent topological corner, edge and bulk states, respectively, and four topological corner modes exist in the eigen-spectrum of the designed structure. We also examine the *Q* factors of these eigenmode, as shown in Figure 4(c). The values of *Q* factor of the corner states could reach as high as 1.1 × 10^4^, which are one order of magnitude higher than those of the bulk and edge states, verifying localization of the topological corner states. Note that, the slight differences in eigen-frequency and *Q* factor between the four corner states could be mainly attributed to the inconsistence between the rotational symmetries of the square structure and the triangular lattice. The electric field distributions corresponding to the four corner states are also shown in Figure 4(d), exhibiting confinement around the corners.

Inspired by the previous literature [29], these corner states could be regarded as topological cavity with less restrictions for lattice type and corner shape. This feature is beneficial for the enhancement of optical nonlinear processes, such as lasing and frequency conversion. As proofs-of-concept, the SHG and THG from the bulk, edge, and corner states are numerically investigated. The optical responses of the designed structure are calculated with a time-varying point excitation, which is written as $E\left(\omega \right)=E_{0}\sin\left(\omega t\right)$ . *E*_0_ = 3 × 10^9^ V/m is the peak intensity of the excitation and *ω* = 2*πf*, where *f* is the frequency of the excitation. We set *f* = 120.02, 123.38, and 124.51 THz, corresponding to bulk, edge, and corner states, respectively.

Figure 4(e) plots the SHG frequency spectra which are obtained from the time spectrum by a band-passing filter and Fourier transformation. The blue, green and red solid curves are extracted from the constructed structure when the bulk, edge and corner states are excited, respectively. The positions of the excitation and detection are optimized to achieve maximum value in each case. It could be easily observed that SHG is significantly enhanced from the corner state. While, the obtained frequency spectra from both bulk and edge states presents no obvious SHG signal. Figure 4(f) plots the electric field distributions of SHG corresponding to bulk, edge, and corner states (the upper-left corner). The SHG from the corner state is strongly confined to a small volume near the upper-left corner due to the localization of the corner state, in contrast, the SHG field corresponding to the bulk and edge states scatters to the volume and propagates along the interfaces, respectively. These results prove that the enhancement of SHG from the corner state relies on the existence of phase vortex, which is liberated from the spatial symmetry of the underlying lattice.

Similarly, the enhancement of THG from the corner state in comparison to the bulk and edge states is investigated and shown in Figure 4(g) and (h). Due to the strongly localized corner state, the THG field is also concentrated within a small volume near the upper-left corner, presenting behaviors which are totally different with the THG from the bulk and edge states. In simple words, the phase vortex introduced by Kekulé modulation ensures the existence of corner state and the enhancement of nonlinear harmonic generation.

Moreover, we build triangular, quadrangular, quinquangular HOTIs based on the above Kekulé modulated lattice (see the Supplementary Material). The existence of corner states in these arbitray shaped HOTIs and the enhanced nonlinear harmonic generation from them additionally demonstrate the advantage of the proposed method.

**Figure 5:**
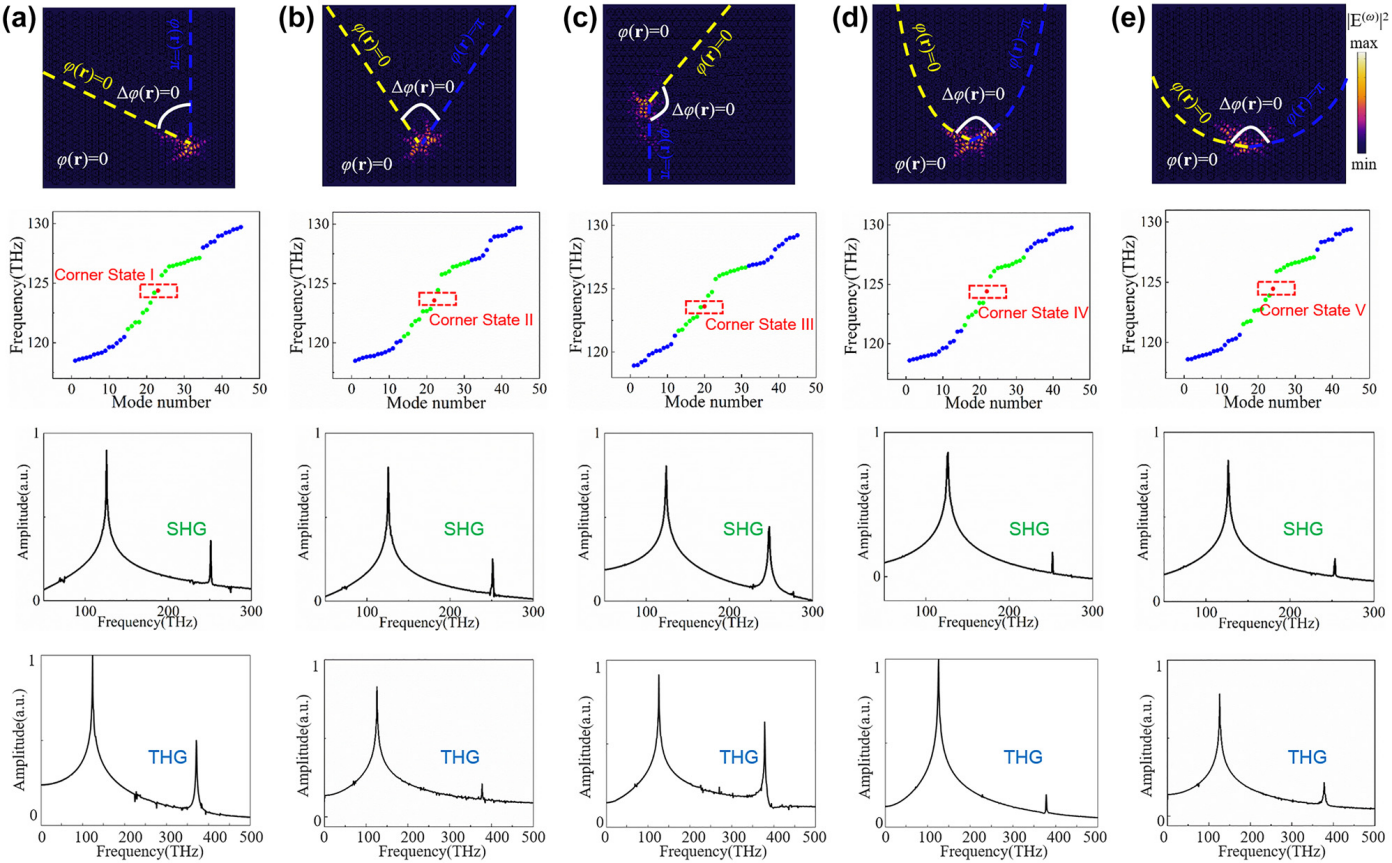
Topological corner states based on Dirac vortex in semi-enclosed domains and enhancement of nonlinear harmonic generation. Results of the corner states labeled as (a) I, (b) II, (c) III, (d) IV, (e) V. First panel: electric field distributions at the corner states. The yellow and blue dashed line marks the boundaries of the Kekulé modulated structure with *φ*(**r**) = 0 and *φ*(**r**) = 2*π*, respectively. The corners with a Dirac vortex of 2*π*-phase winding are outlined by the white solid lines. Second panel: eigenmode results of the semi-enclosed domains. Third panel: SHG frequency spectra obtained from the corner states. Fourth panel: THG frequency spectra obtained from the corner states.

Besides these enclosed domains, the corners where the phase vortex exists in semi-enclosed domains can likewise support topological corner states, even the corners are formed with curved boundaries. Figure 5 presents the simulation results of five corner states formed in semi-enclosed domains. The region satisfying the two-dimensional Laplace’s equation $\Delta \varphi \left(r\right)=0$ is constructed with aperiodic Kekulé lattice, and the surrounding region is constructed with the lattice whose modulation phase is $\varphi \left(r\right)=0$ . The boundaries between these two regions form a corner with phase vortex. The electric field distributions of the corner states are shown in the first panel of Figure 5, exhibiting strong field confinement around the corners. The eigenmode results shown in the second panel of Figure 5 demonstrate the existence of corner states. Note that the frequency of the corner states maintains around 124 ∼ 125 THz even though their shapes are significantly different, proving the topological robustness of the corner state from one aspect. Therefore, enhanced SHG and THG could be obtained from these various shaped corner states, which are completely unrestricted by the symmetry of the underlying lattice. The SHG and THG frequency spectra detected from these corner states are plotted in the third and fourth panels of Figure 5, respectively, presenting significant enhancement of SHG and THG from the corner states.

To further demonstrate the advantage, we calculate the quality factors of the corner states and the efficiencies of both SHG and THG from the corner states in Figure 5. Herein, the SHG and THG efficiencies are defined as $\eta_{\text{SHG}}=P_{\text{SH}}/P_{\text{FF}}^{2}$ and $\eta_{\text{THG}}=P_{\text{TH}}/P_{\text{FF}}^{3}$ , respectively. *P*_SH_, *P*_TH_, and *P*_FF_ represent the power of second, third harmonic and fundamental frequency wave, respectively. The calculated results are summarized in Table 1. All these corner states have high quality factors ranging from 5.5 × 10^3^ to 1.2 × 10^4^, indicating their great ability to harness fundamental field and generate nonlinear fields. In addition, the efficiencies of SHG from the corner states I, II, III, IV, and V are calculated to be 7.0 × 10^−3^ W^−1^, 6.6 × 10^−3^ W^−1^, 9.7 × 10^−3^ W^−1^, 7.8 × 10^−3^ W^−1^, and 8.4 × 10^−3^ W^−1^, respectively. Meanwhile, the efficiencies of THG from the corner states I, II, III, IV, and V are calculated to be 2.0 × 10^−4^ W^−2^, 1.6 × 10^−4^ W^−2^, 3.7 × 10^−4^ W^−2^, 2.2 × 10^−4^ W^−2^, and 3.4 × 10^−4^ W^−2^, respectively. The efficiencies of the obtained nonlinear harmonic generation are comparable to those observed in both topological resonances [42], [45] and a single bound state in the continuum resonance [63].

**Table 1:** Quality factors of the various corner states in Figure 5, and the efficiencies of the corresponding SHG and THG.

Corner state	*Q*-factor	SHG	THG
I	6.9	7.0	2.0
II	5.5	6.6	1.6
III	12	9.7	3.7
IV	8.4	7.8	2.2
V	9.8	8.4	3.4
Units	×10^3^	×10^−3^ W^−1^	×10^−4^ W^−2^

**Figure 6:**
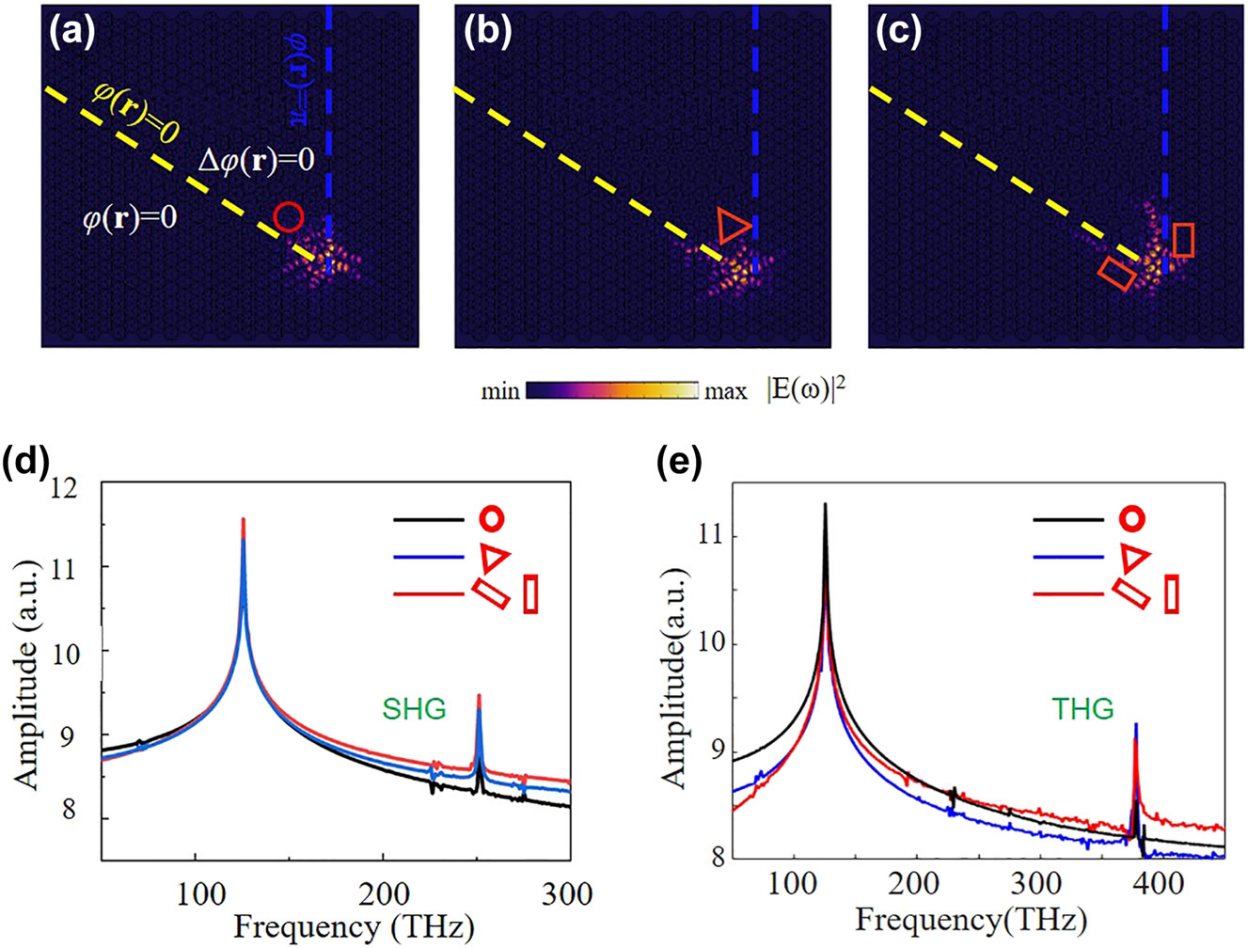
Topological robustness of the designed corner state and SHG, THG from the corner state. Electric field distributions at the corner states when dielectric materials within the (a) circle, (b) triangle, and (c) rectangles are removed. (d) SHG and (e) THG frequency spectrum obtained from the corner states when structure defects exist. The yellow and blue dashed line marks the boundaries of the Kekulé modulated structure with *φ*(**r**) = 0 and *φ*(**r**) = 2*π*, respectively.

The topological robustness of the proposed corner states based on the Dirac vortex and the nonlinear harmonic generation from them are investigated by introducing several structural defects. Figure 6(a)–(c) plot the electric field distributions at the corner states when the dielectric materials with the red boxes are removed. Compared to the results without defect in Figure 5(a), the electric field distribution maintains its profile well, presenting strong confinement of electric field at the corners. Figure 6(d) and (e) plot the SHG and THG frequency spectrum, respectively, obtained from the corner states when these structural defects exist. It is clearly shown that the enhanced SHG and THG from these three corner states are spectrally overlapped. It is because the topological states are protected by the global character of the system, making the local defects almost have no influence on the electric field distribution. In a word, the SHG and THG processes from these three corner states have similar performance. Therefore, these results demonstrate the advantage of the topological robustness of the designed corner states against the structural defects, and the stability of the SHG and THG from the corner states.

As a closing remark, we would like to talk about the experimental feasibility of this study. The preparation of the designed structure in experiment could be possible with the advanced fabrication technology, such as patterning photoresist, reactive ion etching, and so on [64]. To excite the corner state, a tunable continuous-wave laser source can be collimated and focused around the corner. The frequency spectra could be collected by a microscope objective, and the fundamental and nonlinear harmonic generation signals could be separated by a filter.

## 3 Conclusions

In this work, we use Kekulé modulation to introduce phase vortices of 2*π* at the corner of topological insulators to construct HOTIs and topological corner state that does not rely on the symmetry of underlying lattice. The nonlinear effect is enhanced by the topological corner state, which is embodied in enhancement the efficiencies of nonlinear harmonic generation of the incident fundamental wave. By investigating various shaped corner states based on the Dirac vortex, including the quality factor and the efficiencies of both SHG and THG from them, it can be concluded that the nonlinear harmonic generation is significantly enhanced from these corner states with unrestricted shapes. Finally, the robustness of the corner states and the nonlinear harmonic generation are tested by introducing structural defects around the corners. Our results may lay the foundation for its future application in integrated optical devices.

## Supplementary Material

Supplementary Material Details

## References

[1] Xiao D., Chang M. C., Niu Q. (2010). Berry phase effects on electronic properties. *Rev. Mod. Phys.*.

[2] Hasan M. Z., Kane C. L. (2010). Colloquium: topological insulators. *Rev. Mod. Phys.*.

[3] Qi X. L., Zhang S. C. (2011). Topological insulators and superconductors. *Rev. Mod. Phys.*.

[4] Haldane F. D. M., Raghu S. (2008). Possible realization of directional optical waveguides in photonic crystals with broken time-reversal symmetry. *Phys. Rev. Lett.*.

[5] Wang Z., Chong Y., Joannopoulos J. D., Soljačić M. (2009). Observation of undirectional backscattering-immune topological electromagnetic states. *Nature*.

[6] Albert V. V., Glazman L. I., Jiang L. (2015). Topological properties of linear circuit lattices. *Phys. Rev. Lett.*.

[7] Wang P., Lu L., Bertoldi K. (2015). Topological phononic crystals with one-way elastic edge waves. *Phys. Rev. Lett.*.

[8] Xia B. Z. (2017). Topological phononic insulator with robust pseudospin-dependent transport. *Phys. Rev. B*.

[9] Chen Z. G., Wu Y. (2016). Tunable topological phononic crystals. *Phys. Rev. Appl.*.

[10] Yu S. Y. (2018). Elastic pseudospin transport for integratable topological phononic circuits. *Nat. Commun.*.

[11] Tian Z. (2020). Dispersion tuning and route reconfiguration of acoustic waves in valley topological phononic crystals. *Nat. Commun.*.

[12] Mousavi S. H., Khanikaev A. B., Wang Z. (2015). Topologically protected elastic waves in phononic metamaterials. *Nat. Commun.*.

[13] Ma G. C., Xiao M., Chan C. T. (2019). Topological phases in acoustic and mechanical systems. *Nat. Rev. Phys.*.

[14] Fleury R., Khanikaev A. B., Alù A. (2016). Floquet topological insulators for sound. *Nat. Commun.*.

[15] Lu L., Joannopoulos J. D., Soljačić M. (2014). Topological photonics. *Nat. Phys.*.

[16] Ozawa T. (2019). Topological photonics. *Rev. Mod. Phys.*.

[17] Khanikaev A. B., Shvets G. (2017). Two-dimensional topological photonics. *Nat. Photonics*.

[18] Wang H. F., Gupta S. K., Xie B. Y., Lu M. H. (2020). Topological photonic crystals: a review. *Front. Optoelectron.*.

[19] Wu L. H., Hu X. (2015). Scheme for achieving a topological photonic crystal by using dielectric material. *Phys. Rev. Lett.*.

[20] Li Y. D. (2022). Effective Hamiltonian for photonic topological insulator with non-Hermitian domain walls. *Phys. Rev. Lett.*.

[21] Yang Y. H. (2019). Realization of a three-dimensional photonic topological insulator. *Nature*.

[22] Chen M. J. (2014). Experimental realization of photonic topological insulator in a uniaxial metacrystal waveguide. *Nat. Commun.*.

[23] Xie B. Y. (2018). Second-order photonic topological insulator with corner states. *Phys. Rev. B*.

[24] Chen X. D., Deng W. M., Shi F. L., Zhao F. L., Chen M., Dong J. W. (2019). Direct observation of corner states in second-order topological photonic crystal slabs. *Phys. Rev. Lett.*.

[25] Dutt A., Minkov M., Williamson I. A. D., Fan S. H. (2020). Higher-order topological insulators in synthetic dimensions. *Light Sci. Appl.*.

[26] Li M. Y. (2020). Higher-order topological states in photonic Kagome crystals with long-range interactions. *Nat. Photonics*.

[27] Luo X. W., Zhang C. W. (2019). Higher-order topological corner states induced by gain and loss. *Phys. Rev. Lett.*.

[28] Xie B. Y. (2019). Visualization of higher-order topological insulating phases in two-dimensional dielectric photonic crystals. *Phys. Rev. Lett.*.

[29] Ota Y. (2019). Photonic crystal nanocavity based on a topological corner state. *Optica*.

[30] Yang L. C., Li G. R., Gao X. M., Lu L. (2022). Topological-cavity surface-emitting laser. *Nat. Photonics*.

[31] Wang X. (2022). Non-Hermitian high-quality-factor topological photonic crystal cavity. *Phys. Rev. A*.

[32] Shi A. (2021). Coupled cavity-waveguide based on topological corner state and edge state. *Opt. Lett.*.

[33] Xin X. (2020). Cavity quantum electrodynamics with second-order topological corner state. *Laser Photon. Rev.*.

[34] Kruk S. S., Gao W. L., Choi D. Y., Zentgraf T., Zhang S., Kivshar Y. (2021). Nonlinear imaging of nanoscale topological corner states. *Nano Lett.*.

[35] Vakulenko A. (2021). Near-field characterization of higher-order topological photonic states at optical frequencies. *Adv. Mater.*.

[36] Smirnova D., Kruk S., Leykam D., Melik-Gaykazyan E., Choi D. T., Kivshar Y. (2019). Third-harmonic generation in photonic topological metasurfaces. *Phys. Rev. Lett.*.

[37] He X. T. (2021). In-plane excitation of a topological nanophotonic corner state at telecom wavelengths in a cross-coupled cavity. *Photon. Res.*.

[38] Sergey S. (2019). Nonlinear light generation in topological nanostructures. *Nat. Nanotechnol.*.

[39] Om K. K., Kim K. H. (2022). Second-harmonic generation based on the dual-band second-order topological corner states. *Phys. Status Solidi Rapid Res. Lett.*.

[40] Om K. K., Kim K. H. (2023). Dual-band higher-order topological states and four-wave mixing in plasmonic valley-Hall metasurfaces. *Phys. Lett. A*.

[41] Ma J., Guo K., Chen F. J., Zhou K. Y., Liu S. T., Guo Z. Y. (2021). Manipulating second harmonic generation in higher-order topological photonic crystals. *Ann. Phys.*.

[42] Guo K., Wu J. T., Chen F. J., Zhou K. Y., Liu S. T., Guo Z. Y. (2021). Second harmonic generation enhancement and directional emission from topological corner state based on the quantum spin Hall effect. *Opt. Express*.

[43] Smirnova D., Leykam D., Chong Y. D., Kivshar Y. (2020). Nonlinear topological photonics. *Appl. Phys. Rev.*.

[44] Lan Z. H., You J. W., Ren Q., Sha W. E. I., Panoiu N. C. (2021). Second-harmonic generation via double topological valley-Hall kink modes in all-dielectric photonic crystals. *Phys. Rev. A*.

[45] Chen Y. F., Lan Z. H., Li J. S., Zhu J. (2021). Topologically protected second harmonic generation via doubly resonant high-order photonic modes. *Phys. Rev. B*.

[46] Yuan Q. C., Gu L. P., Fang L., Gan X. T., Chen Z. G., Zhao J. L. (2022). Giant enhancement of nonlinear harmonic generation in a silicon topological photonic crystal nanocavity chain. *Laser Photon. Rev.*.

[47] Zhou H. P. (2022). Controllable second harmonic generation based on topological spin-dependent edge states. *J. Appl. Phys.*.

[48] He L., Addison Z., Eugene J. M., Zhen B. (2020). Quadrupole topological photonic crystals. *Nat. Commun.*.

[49] Liu Y. H., Wang Y. Z., Hu N. C., Lin J. Y., Lee C. H., Zhang X. (2020). Topological corner modes in a brick lattice with nonsymmorphic symmetry. *Phys. Rev. B*.

[50] Hou C. Y., Chamon C., Mudry C. (2007). Electron fractionalization in two-dimensional graphenelike structures. *Phys. Rev. Lett.*.

[51] Gao X. M. (2020). Dirac-vortex topological cavities. *Nat. Nanotechnol.*.

[52] Gao P. L., Torrent D., Cervera F., San-Jose P., Sánchez-Dehesa J., Christensen J. (2019). Majorana-like zero modes in Kekulé. *Phys. Rev. Lett.*.

[53] Wu X. X., Meng Y., Hao Y. R., Zhang R. Y., Li J. S., Zhang X. (2021). Topological corner modes induced by Dirac vortices in arbitrary geometry. *Phys. Rev. Lett.*.

[54] Wei G. C., Liu Z. Z., Wang L. C., Song J. Y., Xiao J. J. (2022). Coexisting valley and pseudo-spin topological edge states in photonic topological insulators made of distorted Kekulé lattices. *Photon. Res.*.

[55] Lu J. Y., Qiu C. Y., Ke M. Z., Liu Z. Y. (2016). Valley vortex states in sonic crystals. *Phys. Rev. Lett.*.

[56] Wu X. (2017). Direct observation of valley-polarized topological edge states in designer surface plasmon crystals. *Nat. Commun.*.

[57] Zhang X. J. (2020). Symmetry-protected hierarchy of anomalous multipole topological band gaps in nonsymmorphic metacrystals. *Nat. Commun.*.

[58] Chamon C. (2000). Solitons in carbon nanotubes. *Phys. Rev. B*.

[59] Sergey S. (2017). Nonlinear optical magnetism revealed by second-harmonic generation in nanoantennas. *Nano Lett.*.

[60] Sautter J. D. (2019). Tailoring second-harmonic emission from (111)-GaAs nanoantennas. *Nano Lett.*.

[61] Rodríguez-Suné L., Trull J., Scalora M., Vilaseca R., Cojocaru C. (2019). Harmonic generation in the opaque region of GaAs: the role of the surface and magnetic nonlinearities. *Opt. Express*.

[62] Mobini E., Espinosa D. H. G., Vyas K., Dolgaleva K. (2022). AlGaAs nonlinear integrated photonics. *Micromachine*.

[63] Carletti L., Koshelev K., Angelis C. D., Kivshar Y. (2018). Giant nonlinear response at the nanoscale driven by bound states in the continuum. *Phys. Rev. Lett.*.

[64] Yang Y. H. (2020). Terahertz topological photonics for on-chip communication. *Nat. Photonics*.

