# Supplementary material for: Shape unrestricted topological corner state based on Kekulé modulation and enhanced nonlinear harmonic generation
Source: Nanophotonics. 2024 Jun 3;13(18):3485–94. doi: 10.1515/nanoph-2024-0116 (PMC11501897; doi:10.1515/nanoph-2024-0116)
Supplement: Supplementary file 1 — Supplementary Material Details [file j_nanoph-2024-0116_suppl_001.docx]

**SUPPLEMENTAL MATERIAL**

Here the Dirac vortex based phtotonic higher-order topological insulators (HOTIs) with more complex shapes, the existence of corner states, and the enhancement of nonlinear harmonic generation are demonstrated.

Firstly, we constructed a triangular HOTI where inner domain is fulfilled with Kekulé modulated lattices (Δ*φ*(**r**)=0) and surrounded by ordinary lattices (*φ*(**r**)=0). Figure S1 shows the simulation results of the triangular HOTI. Unsurprisingly, there exist three topological corner states (Fig. S1(a)) and their Q-factors are much higher than the bulk and edge states (Fig. S1(b)). The electric field distributions of these corner states are all showing strong confinement near the corners (Fig. S1(c)), indicating the high quality of these corner states and the great ability to improve the nonlinear optical processes. Figures S1(d) and S1(e) plot the SHG and THG frequency spectrum which are obtained from the top corner. As expected, the nonlinear harmonic generation could be enhanced significantly from the corner state.

*
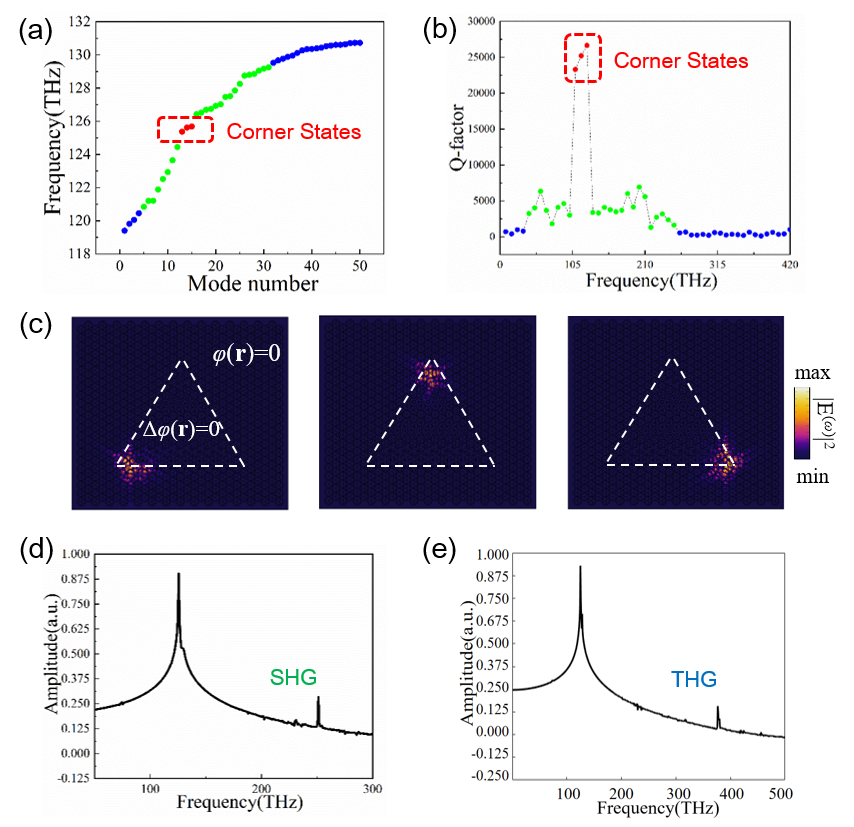
*

Fig. S1 A triangular HOTI based on the Dirac vortex. (a) Eigenmodes of the designed triangular HOTI. (b) Q factor of the eigenmodes. (c) Electric field distributions of the corner states which are topologically protected by the Dirac vortex. The white dashed lines mark the boundaries between the Kekulé modulated domain and the ordinary domain. (d) SHG and (e) THG frequency spectrum obtained from the top corner.

Secondly, we constructed a quadrangular HOTI where inner domain is fulfilled with Kekulé modulated lattices (Δ*φ*(**r**)=0) and surrounded by ordinary lattices (*φ*(**r**)=0). Figure S2 shows the simulation results of the quadrangular HOTI. Similarly, there exist four topological corner states (Fig. S2(a)) and their Q-factors are higher than the bulk and edge states (Fig. S2(b)). The electric field distributions of these corner states are showing strong confinement near the corners (Fig. S2(c)), indicating the high quality of these corner states and their great ability to improve the nonlinear optical processes. Figures S2(d) and S2(e) plot the SHG and THG frequency spectrum which are obtained from the bottom-left corner. As expected, the nonlinear harmonic generation could be enhanced significantly from the corner state.


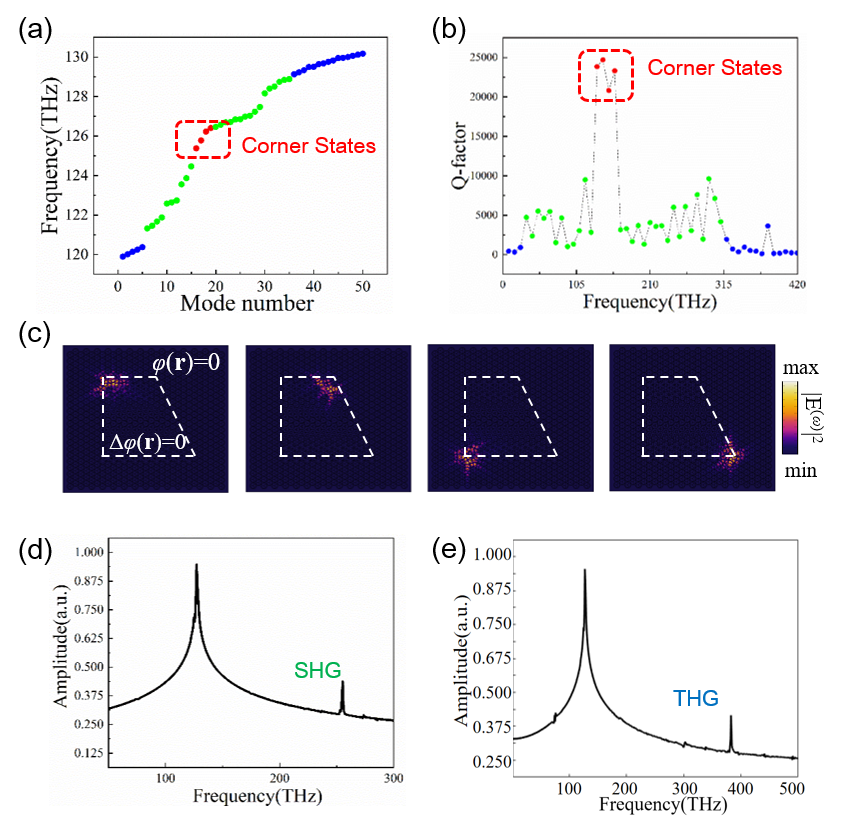


Fig. S2 A quadrangular HOTI based on the Dirac vortex. (a) Eigenmodes of the designed quadrangular HOTI. (b) Q factor of the eigenmodes. (c) Electric field distributions of the corner states which are topologically protected by the Dirac vortex. The white dashed lines mark the boundaries between the Kekulé modulated domain and the ordinary domain. (d) SHG and (e) THG frequency spectrum obtained from the bottom-left corner.

Thirdly, we constructed a quinquangular HOTI where inner domain is fulfilled with Kekulé modulated lattices (Δ*φ*(**r**)=0) and surrounded by ordinary lattices (*φ*(**r**)=0). Figure S3 shows the simulation results of the quinquangular HOTI. Similarly, there exist five topological corner states (Fig. S3(a)) and their Q-factors are higher than the bulk and edge states (Fig. S3(b)). The electric field distributions of these corner states are showing strong confinement near the corners (Fig. S3(c)), indicating the high quality of these corner states and their great ability to improve the nonlinear optical processes. Figures S3(d) and S3(e) plot the SHG and THG frequency spectrum which are obtained from the bottom-right corner. As expected, the nonlinear harmonic generation could be enhanced significantly from the arbitrarily chosen corner state.


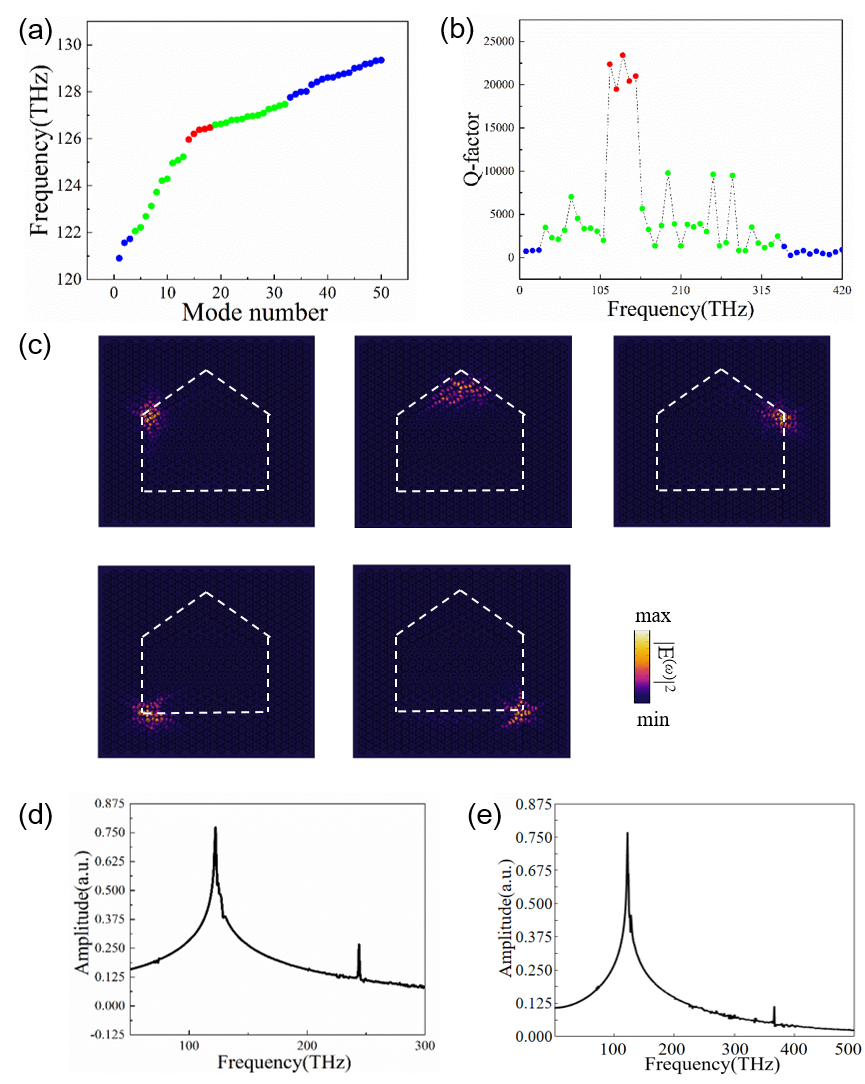


Fig. S3 A quinquangular HOTI based on the Dirac vortex. (a) Eigenmodes of the designed quinquangular HOTI. (b) Q factor of the eigenmodes. (c) Electric field distributions of the corner states which are topologically protected by the Dirac vortex. The white dashed lines mark the boundaries between the Kekulé modulated domain and the ordinary domain. (d) SHG and (e) THG frequency spectrum obtained from the bottom-right corner.
